# Duodenal neuroendocrine tumor: A rare case report

**DOI:** 10.1097/MD.0000000000024635

**Published:** 2021-02-12

**Authors:** Xuewen Wang, Yanbin Wu, Xuefeng Cao, Xingyuan Zhang, Yu Cheng, Lingqun Kong

**Affiliations:** a Department of Hepatobiliary Surgery, Binzhou Medical University Hospital, Binzhou City; b Department of Hepatobiliary Surgery, Yantai Affiliated Hospital of Binzhou Medical University, Yantai City, Shandong Province, People's Republic of China.

**Keywords:** case report, neuroendocrine tumor

## Abstract

**Rationale:**

Duodenal neuroendocrine tumor (d-NET) is a rare tumor originating in the neuroendocrine system. The clinical manifestations of d-NET are similar to those of other digestive tract tumors, resulting in a lack of specificity and complex clinical symptoms.

**Patient concerns:**

A 55-year-old female patient was admitted to our hospital with a chief complaint of an abdominal mass that had been present for more than 4 months.

**Diagnoses:**

The upper abdomen enhanced computed tomography scan showed an uneven density mass across the upper abdomen, and the tumor size was approximately 6.2 × 5.8 cm with obvious visible enhancement present in 1 area and a cystic nonenhanced area. The postoperative pathology showed the tumor cells to be positive for chromogranin, synaptophysin, cytokeratin, CD56 (partial weak), negative for vimentin, CD117, DOG-1, CD34, S-100, SMA, desmin, and Ki-67 approximately 2%, which confirmed the diagnosis of d-NETs.

**Interventions:**

We preferred laparoscopic surgical exploration, but the tumor started at the ascending part of the duodenum and involved the mesenteric artery. As the branches of the superior mesenteric artery were intertwined with the tumor, it was difficult to operate with the endoscope, so we converted to open laparotomy. The postoperative pathology revealed the presence of d-NET.

**Outcomes:**

The patient recovered uneventfully and was discharged after the operation. One-month and 3-month follow-up after surgery, showed no evidence of recurrence.

**Lessons:**

Radiological imaging studies are insufficient for the differential diagnosis of abdominal mass from other diseases, whereas surgery is the only radical treatment method, and the preferred surgical method is still active radical resection of the tumor.

## 1 Introduction

Neuroendocrine tumor (NET) is s a type of tumor refers to the body's neuroendocrine tumor cells and originates from peptide neurons and neuroendocrine cells, its nature is not entirely malignant potential. NET is a rare disease, its less than 1% in all malignant tumors, but can be present in many organs and tissues. Gastrointestinal neuroendocrine tumor is a rare type of special low-grade malignant tumor, that can occur in any part of the digestive tract, such as the stomach and rectum, accounting for about 0.4% to 1.8% of all gastrointestinal malignancies.^[1]^ Duodenal neuroendocrine tumor (d-NET) is relatively rare tumor and only accounts for 2% to 3% of gastrointestinal neuroendocrine tumors.

## 2 Case presentation

### 2.1 Patient information

The patient, a 55-year-old female, was admitted in the hospital on July 5, 2019, because of the discovery of an abdominal mass that had been present for more than 4 months. Approximately 4 months prior, the patient inadvertently found a hard upper abdomen mass but reported no associated pain. The patient self-reported the tumor to be about the size of an egg but reported no nausea, vomiting, abdominal pain, diarrhea, haematemesis, blood in the stool or other discomfort. A local hospital computed tomography (CT) enhancement scan revealed a solid cystic mass in the upper abdomen, and the initial segment of the jejunum was unclear, suggesting the possibility of a stromal tumor. The patient was physically healthy. The examination upon admission revealed a soft abdomen that allowed the abdominal mass to be palpated. The mass was hard and appeared to contact the upper left abdominal area, and the size was approximately 5 × 6 × 5 cm. The patient's level of activity was good, and there was no tenderness. After admission, the relevant examinations were completed, and no obvious abnormalities were found in the laboratory tests, which included routine blood tests, biochemistry tests, and tumor marker analyses.

### 2.2 Imaging findings

**Figure 1:**
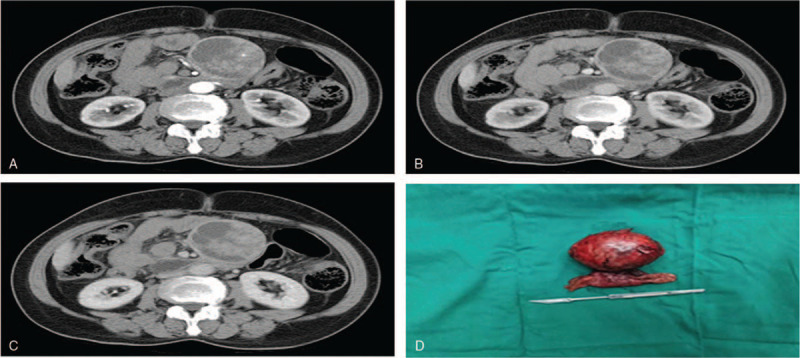
Contrast-enhanced CT scan showing a mass in the abdominal cavity (A–C) and. Photographs of the tumor (D) arterial phase; (B) venous phase; (C) lag phase; (D) completely resected surgical specimen. CT = computed tomography.

The upper abdomen enhanced CT showed the following: an abnormally dense shadow was present across the upper abdomen, but the side was clear, and the tumor size was approximately 6.2 × 5.8 cm. There was an uneven density within the lesion, with obvious visible enhancement present in 1 area and a cystic nonenhanced area. The lesion was closely related to the intestinal tract of the small intestine (beginning approximately at the jejunum), and a small arterial branch was seen inside. There were no obviously enlarged lymph nodes beyond the retroperitoneum (Fig. 1A–C).

### 2.3 Therapeutic interventions and histopathological findings

**Figure 2:**
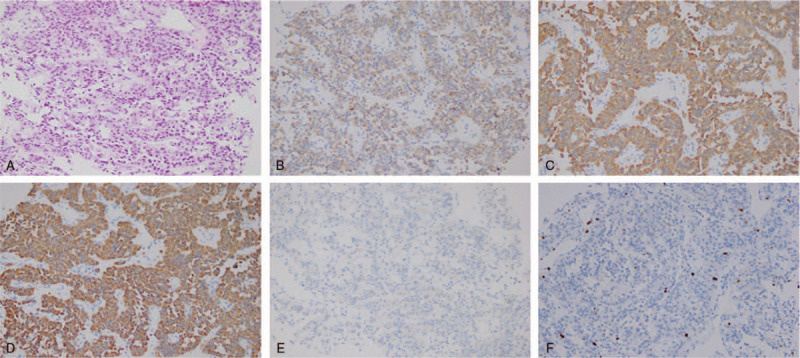
Immunohistochemistry findings of ultrasound-guided abdominal biopsy (A–F) Hematoxylin-eosin (HE) staining (×200); Tumor cells are positivity for CgA (B), Syn (C), and CK (D), but negative for CD56 (E) (×200); (F) Ki-67 positive rate <1% (×200). CK = cytokeratin.

**Figure 3:**
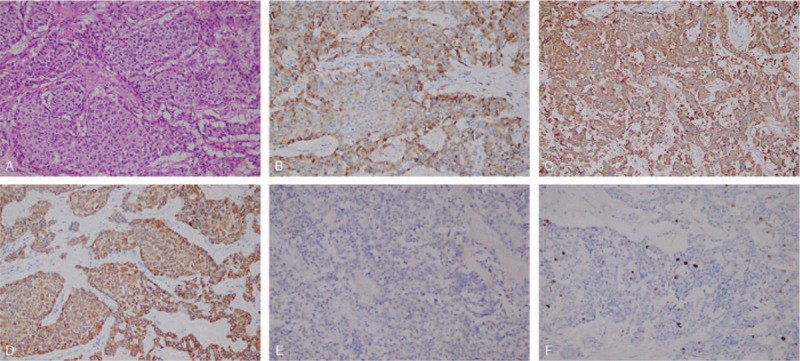
Immunohistochemistry findings of postoperative specimen (A–F). Hematoxylin-eosin (HE) staining (×200); tumor cells are positivity for CgA (B), Syn (C), and CK (D), but weakly positive for CD56 (E) (×200); (F) Ki-67 positive rate approximately 2% (×200). CgA = chromogranin.

First, the patient underwent ultrasound-guided abdominal biopsy, and the postoperative pathology results showed the following: the cells showed alveolar and trabecular growth and were of a consistent shape, with round or oval nuclei and visible nuclear inclusion bodies. No clear mitotic figures were found. The results of immunohistochemical marker staining were as follows: chromogranin A (CgA)+, synaptophysin (Syn)+, cytokeratin +, vimentin–, CD56-, β-catenin membrane+, CD10–, PR–, CD117–, CD34–, CD7+, CD20–, CR–, and Ki-67 positive rate <1% (Fig. 2A–F). Considering the pathology and immunohistochemistry results together, NET was considered, with a suspicion of neuroendocrine tumor G1. The patient was diagnosed with d-NET. After surgical contraindications were excluded, laparoscopic-assisted partial duodenal resection was performed under general anesthesia on July 17, 2019. The operation was successful, and the tumor was completely removed (Fig. 1D). The specimen was sent for pathology testing. The postoperative pathology results showed the following: an intestinal NET (G2) invading the serosa, and the surgical margin was not affected. The postoperative immunohistochemistry results were as follows: CgA+, Syn+, cytokeratin +, vimentin–, CD56 partial weak+, CD117–, DOG-1–, CD34–, S-100–, SMA–, desmin–, and Ki-67 approximately 2% (Fig. 3A–F). A pathological stage of WHO pT2NX was assigned.

### 2.4 Follow-up and outcomes

After the operation, gastrointestinal decompression, a limited diet, acid suppression, anti-infection strategies, and fluid replacement were employed. The patient's condition gradually improved, and she was discharged. One-month and 3-month on follow-up after surgery, showed no evidence of recurrence.

## 3 Discussion

Neuroendocrine neoplasm (NET) is a type of tumor that originates from stem cells and has neuroendocrine markers that produce bioactive amine or peptide hormones that cause different clinical syndromes, for example, carcinoid syndrome, has a wide range of malignant potential.^[2,3]^ and can occur in a variety of organs and tissues throughout the body, with the most common location being the digestive system, which accounts for approximately 50.6% of NETs.^[4]^ However, NETs also occur in the duodenum and less commonly in the duodenal bulb and descending segments, but the incidence is low, accounting for only 4% of all digestive tract tumors.^[5]^ The clinical manifestations of NETs are similar to those of other digestive tract tumors, resulting in a lack of specificity and complex clinical symptoms.^[6]^ There are relatively few studies on d-NETs in China, but their incidence and prevalence are significantly increasing. The reasons may be related to difficulties associated with imaging examination and pathological diagnosis. The increasing levels of incidence and prevalence and the extensive promotion and application of gastrointestinal cancer screening are closely related.^[7,8]^ Imaging examination can be used to determine the general source of the tumor, but the specific type of the tumor cannot be determined. Only histopathology can be used to judge the tumor type. In this case, colonoscopy and CT were performed preoperatively, and involvement of the duodenum was identified. No swelling was found. With invasion of surrounding tissues, preoperative routine pathology is the only way to determine the disease and is also the basis for tumor classification. The preoperative histopathological examination of this patient suggested d-NET. The main points of pathological diagnosis of NETs include several aspects: first, whether the tumor stains positive for the NET markers Syn and CgA by immunostaining.^[9]^ Next, the tumor proliferative activity clarifies the grade of the tumor, and the proliferative activity of the tumor is evaluated by the number of mitotic figures or the Ki-67 index. According to the number of mitotic figures in the tumor cells and the Ki-67 index, NETs are classified into 3 grades: G1, the number of mitotic figures is <2 and/or the Ki-67 index is <2%; G2, the number of mitotic figures is 2 to 20 and/or the Ki-67 index is 3% to 20%; and G3, the number of mitotic figures is >20 and/or the Ki-67 index is >20%.G1 and G2 tumors are NETs, and G3 tumors are neuroendocrine carcinomas.^[10]^ The preoperative pathology suggested that the tumor was in the G1 phase, so early surgery was required. Laparoscopic duodenal partial resection was performed as the surgical procedure. However, the tumor started at the ascending part of the duodenum and involved the mesenteric artery. The inferior mesenteric vein and the pancreatic uncinate process are closely related. As the branches of the superior mesenteric artery were intertwined with the tumor, it was difficult to operate with the endoscope, so we convert to open laparotomy. Part of the duodenum including the tumor and part of the jejunum were completely removed, and the horizontal part of the duodenum was anastomosed with the end of the jejunum. The excised specimens were sent for pathology testing, and the pathological results were basically consistent with the preoperative histopathology results, which further confirmed that the patient had d-NET.

There are few reports on the diagnosis and treatment of d-NETs, and d-NETs generally have good differentiation, slow growth, and good prognosis. However, some tumors display the opposite characteristics.^[11]^ The prognosis of this disease may be related to the pathological type, staging, endocrine components, postoperative adjuvant therapy, and other factors. Lymph node metastasis and distant metastasis are independent risk factors for poor prognosis of NETs.^[12]^ In short, d-NETs has an insidious onset, and clinical symptoms are not specific, especially in the early stage. In addition, there are no obvious symptoms, and the symptoms are not taken seriously. After progression, patients may have symptoms such as upper abdominal pain, fatigue, weight loss, digestive tract bleeding, abdominal mass, and anemia. At this time, the opportunity for radical surgery is often lost, so early diagnosis is particularly important. Thorough preoperative examination, detailed surgical evaluation, and early surgical treatment can avoid missed diagnoses and misdiagnoses of this disease. Postoperative adjuvant therapy based on pathological staging and immunohistochemistry can prolong the survival time of patients. At present, surgery is the only radical treatment method, and the preferred surgical method is still active radical resection of the tumor.^[13]^ After follow-up, the patient had a good prognosis and no tumor recurrence or metastasis. It has been reported that patients with d-NETs with tumors less than 1 cm without lymph node metastasis can undergo endoscopic local excision and have a better therapeutic effect.^[14,15]^ At present, the pathogenesis of d-NETs is not completely clear, and further research is needed. Clinical treatment usually involves surgical resection (radial resection, palliative resection, radiofrequency ablation, and embolization), biological therapy, and targeted therapy in the early and middle stages. Regular physical examination, including gastroscopy, can improve the detection of d-NETs, diagnose the tumor stage early and predict prognosis. Ultimately, the appropriate treatment can improve the patient's 5-year survival rate.

## Author contributions

**Funding acquisition:** Yanbin Wu.

**Investigation:** Xuefeng Cao.

**Supervision:** Xingyuan Zhang.

**Writing – original draft:** Xuewen Wang.

**Writing – review and editing:** Yu Cheng, Lingqun Kong.

## Abbreviations

Abbreviations: CgA = chromogranin, CT = computed tomography, d-NET = duodenal neuroendocrine tumor, NET = neuroendocrine tumor, Syn = synaptophysin.
